# A real-world pharmacovigilance study of FDA adverse event reporting system (FAERS) events for niraparib

**DOI:** 10.1038/s41598-022-23726-4

**Published:** 2022-11-29

**Authors:** Menglin Guo, Yamin Shu, Guosong Chen, Juan Li, Feie Li

**Affiliations:** Department of Pharmacy, Tongji Hospital, Tongji Medical College, Huazhong University of Science and Technology, No. 1095 Jiefang Avenue, Wuhan, 430030 China

**Keywords:** Drug discovery, Health care, Medical research, Oncology

## Abstract

Niraparib was approved for the treatment of platinum-sensitive recurrent epithelial ovarian cancer, fallopian tube and primary peritoneal cancer. The authors retrospectively investigated niraparib-related adverse events (AEs) through data mining of the US Food and Drug Administration Adverse Event Reporting System (FAERS). Four algorithms were employed to quantify the signals of niraparib associated AEs, using data from the FAERS between 2017 and 2021. MYSQL 8.0, Navicat Premium 15, Microsoft EXCEL 2019 and the GraphPad Prism 8 were used to conduct statistical analysis. There are 7,238,157 reports collected from the FAERS database, of which 11,701 reports listed niraparib as the ‘primary suspected (PS)’ drug. A total of 97 significant disproportionality PTs conforming to the four algorithms were simultaneously retained. Unexpected significant AEs such as neuropathy peripheral, photosensitivity reaction, gastrooesophageal reflux disease might also occur. The median onset time of niraparib-associated AEs was 18 days (interquartile range [IQR] 4–66 days), and most of the cases occurred within the first months after niraparib initiation. The study found niraparib-associated AEs and might provide important support for clinical monitoring and risk identification of niraparib.

## Introduction

Niraparib is a potent and highly selective inhibitor of poly (adenosine diphosphate [ADP]–ribose) polymerase (PARP) 1/2 that play a role in DNA repair^1,2^. In vitro studies have shown that niraparib-induced cytotoxicity may involve inhibition of PARP enzymatic activity and increased formation of PARP-DNA complexes resulting in DNA damage, apoptosis, and cell death^3^. Niraparib was approved in the United States Food and Drug Administration (FDA) in March 2017. Maintenance therapy with niraparib can lengthen progression-free survival and the chemotherapy-free interval for adult patients with platinum-sensitive recurrent epithelial ovarian cancer, fallopian tube or primary peritoneal cancer^4–6^. Better understanding of the real-world safety profile of niraparib in patients with ovarian cancer will lead to better compliance, decrease interruptions, and reflect on the desirable progression-free survival and overall survival.

In the clinical phase II and phase III studies of niraparib, the most common treatment-emergent adverse drug reactions (ADRs) included hematological toxicity (white blood cell count decreased, neutrophil count decreased, platelet count decreased, anemia and so on), fatigue, insomnia, hypertension, constipation, nausea, vomiting and diarrhea, which were manageable in the clinical trial setting^7–9^. A study in patients with recurrent ovarian cancer treated with niraparib showed that niraparib was well tolerated, with 14.7% of patients discontinuing treatment due to treatment-emergent adverse events (TEAEs)^10^. The long-term efficacy and safety data of niraparib have only been reported in case reports or clinical trials, and ADRs are mainly focused on a single or several systems due to relatively small sample sizes, selection criteria and limited duration of follow-ups. Currently, data on the large sample and real-world comprehensive safety of niraparib are lacking. Niraparib is the most representative PARP inhibitor in the maintenance therapy of platinum-sensitive advanced/recurrent epithelial ovarian, fallopian tube or primary peritoneal cancer, and has been widely used in clinic^11^. With the wide use of niraparib in clinical practice, and scarcity in evaluations of the adverse events (AEs) in real-world cohorts, we conducted this pharmacovigilance analysis to evaluate the safety profile of niraparib. Therefore, it is very necessary to use data mining algorithm to search for the potential ADRs signals of niraparib by post-marketing monitoring.

FAERS is the FDA Adverse Event Reporting System, which is a public, voluntary, spontaneous reporting database and a post-marketing repository for AE reports, product quality complaints, and medication error reports related to any FDA-approved therapy^12,13^. Manufacturers are required to send any AE reports to the FDA, while global healthcare professionals and consumers may voluntary do so. The spontaneous reporting system has been utilized in pharmacovigilance for safety assessment suspected AEs and plays a major part in signal identification due to inherent limitations of clinical trials such as stringent trial design, strict enrollment criteria, relatively small sample size and limited follow-up duration^11^. In this study, we evaluated the AE reports of niraparib, and aimed to assess the potential relevance between niraparib and AE reports through data mining of FAERS.

## Results

### Descriptive analysis

During the study period, a total of 7,238,157 eligible cases were identified from the quarterly files from FAERS. There were 11,701 case reports of niraparib as the PS and 82,871 AEs were found to be related to niraparib. The clinical characteristics of events with niraparib were described in Table 1. Among all AEs, females (78.55%) accounted for a larger proportion than males (1.79%). In terms of age composition, patients were mainly aged ≥ 40 years old. Ovarian cancer was the most reported indication (70.61%), followed by fallopian tube cancer (4.86%), malignant peritoneal neoplasm (4.54%) and uterine cancer (1.28%). Death or life-threatening events were reported in 771 (6.59%) and 592 (5.06%) cases, respectively. In addition to other serious medical events (46.67%), hospitalization-initial or prolonged (22.34%) was the most frequently reported serious outcome. The high percentage of death or life-threatening events might be more related to disease progression of tumor. Most of AEs were reported from America (87.29%), followed by Japan (2.41%), Canada (1.83%), France (1.57%), and Great Britain (1.43%). Interestingly, consumers represented the main source of reports (77.43%). In terms of reporting years, the most reported year was 2021 (32.92%), followed by 2018 (27.08%), 2020 (20.25%), 2019 (11.79%), and 2017 (7.96%), respectively.

**Table 1:** Clinical characteristics of reports with niraparib from the FAERS database (2017 to 2021).

Characteristics	Case number, n	Case proportion, %
Number of events	11,701	
**Sex**		
Female	9191	78.55
Male	209	1.79
Unknow	2301	19.66
**Age**		
< 40	58	0.50
40–50	253	2.16
> 50	3925	33.54
Unknown or missing	7465	63.80
**Indications (Top four)**		
Ovarian cancer	8262	70.61
Fallopian tube cancer	569	4.86
Malignant peritoneal neoplasm	531	4.54
Uterine cancer	150	1.28
**Serious outcome**		
Death	771	6.59
Disability	30	0.26
Hospitalization-initial or prolonged	2614	22.34
Life-threatening	592	5.06
Other serious medical events	5461	46.67
**Reported countries (Top five)**		
America	10,214	87.29
Japan	282	2.41
Canada	214	1.83
France	184	1.57
Great Britain	167	1.43
**Reported person**		
Consumer	9060	77.43
Health professional	184	1.57
Lawyer	2	0.02
Physician	1634	13.96
Other health-professional	445	3.80
Pharmacist	122	1.04
**Reporting year**		
2017	931	7.96
2018	3169	27.08
2019	1379	11.79
2020	2370	20.25
2021	3852	32.92

Moreover, We perform the sensitivity analysis by excluding consumer reports, to judge whether it will affect the results of signals in Table 3. Although the number of reports decreased in the sensitivity analysis, the signal strength increased significantly (Supplementary Table S1). All the signals in the Table 3 were found in sensitivity analysis results except the PT of Blood count abnormal.

### Signal of system organ class

Signal strengths of reports of niraparib at the System Organ Class (SOC) level are described in Table 2. Statistically, we found that niraparib-induced AEs occurrence targeted 27 organ systems. The significant SOCs that at least one of the four indices met the criteria were general disorders and administration site conditions (SOC: 10018065, 6849), investigations (SOC: 10022891, 6136), gastrointestinal disorders (SOC: 10017947, 6100), nervous system disorders (SOC: 10029205, 4681), injury, poisoning and procedural complications (SOC: 10022117, 4303).

**Table 2:** Signal strength of reports of niraparib at the System Organ Class (SOC) level in FAERS database.

System organ class (SOC)	Niraparib cases reporting SOC	ROR (95% two-side Cl)	PRR (χ^2^)	IC (IC025)	EBGM (EBGM05)
General disorders and administration site conditions	6849	2.23 (2.15–2.31)*	1.51 (1919.11)	0.59 (0.55) *	1.51 (1.45)
Investigations	6136	8.52 (8.21–8.83)*	4.58 (19,236.96) *	2.18 (2.14) *	4.55 (4.39) *
Gastrointestinal disorders	6100	4.93 (4.76–5.11)*	2.88 (9118.11) *	1.52 (1.48) *	2.87 (2.77) *
Nervous system disorders	4681	2.54 (2.45–2.64)*	1.93 (2623.36)	0.94 (0.89) *	1.92 (1.85)
Injury, poisoning and procedural complications	4303	1.40 (1.35–1.46)*	1.25 (312.76)	0.33 (0.28) *	1.25 (1.21)
Psychiatric disorders	3466	2.82 (2.71–2.93)*	2.28 (2855.85) *	1.19 (1.13) *	2.28 (2.19) *
Vascular disorders	3023	1.96 (1.88–2.04)*	1.71 (1044.53)	0.77 (0.71) *	1.71 (1.64)
Cardiac disorders	2679	2.37 (2.27–2.47)*	2.05 (1623.36) *	1.03 (0.97) *	2.05 (1.96)
Respiratory, thoracic and mediastinal disorders	2675	1.77 (1.69–1.85)*	1.59 (687.24)	0.67 (0.61) *	1.59 (1.52)
Musculoskeletal and connective tissue disorders	2557	2.04 (1.95–2.13)*	1.81 (1049.19)	0.85 (0.79) *	1.81 (1.73)
Skin and subcutaneous tissue disorders	2343	1.29 (1.23–1.35)*	1.23 (121.58)	0.30 (0.23) *	1.23 (1.18)
Metabolism and nutrition disorders	1999	2.69 (2.56–2.82)*	2.4 (1750.42) *	1.26 (1.19) *	2.39 (2.28) *
Blood and lymphatic system disorders	1945	3.81 (3.63–4.00)*	3.34 (3340.09) *	1.73 (1.66) *	3.33 (3.17) *
Neoplasms benign, malignant and unspecified (incl cysts and polyps)	1941	2.19 (2.09–2.3)*	1.99 (1045.46)	0.99 (0.92) *	1.99 (1.90)
Infections and infestations	1583	1.25 (1.18–1.32)*	1.21 (67.50)	0.28 (0.20) *	1.21 (1.15)
Surgical and medical procedures	1271	3.54 (3.34–3.75)*	3.26 (2053.40) *	1.70 (1.61) *	3.25 (3.07) *
Renal and urinary disorders	1262	1.60 (1.51–1.70)*	1.54 (255.62)	0.62 (0.53) *	1.54 (1.45)
Reproductive system and breast disorders	677	1.31 (1.21–1.41)*	1.29 (46.14)	0.36 (0.25) *	1.29 (1.19)
Eye disorders	551	1.11 (1.02–1.21)*	1.10 (5.53)	0.14 (0.01) *	1.10 (1.01)
Immune system disorders	500	0.41 (0.38–0.45)	0.44 (397.38)	− 1.19 (− 1.32)	0.44 (0.40)
Hepatobiliary disorders	425	1.36 (1.23–1.49)*	1.34 (38.12)	0.42 (0.28) *	1.34 (1.22)
Social circumstances	287	2.08 (1.85–2.33)*	2.05 (155.59) *	1.02 (0.85) *	2.05 (1.82)
Ear and labyrinth disorders	212	1.42 (1.24–1.62)*	1.41 (25.58)	0.49 (0.29) *	1.41 (1.23)
Endocrine disorders	171	0.55 (0.48–0.64)	0.56 (60.68)	− 0.84 (− 1.06)	0.56 (0.48)
Product issues	91	0.18 (0.14–0.22)	0.18 (347.2)	− 2.45 (− 2.76)	0.18 (0.15)
Congenital, familial and genetic disorders	19	0.28 (0.18–0.43)	0.28 (35.93)	− 1.87 (− 2.53)	0.28 (0.18)
Pregnancy, puerperium and perinatal conditions	13	0.06 (0.03–0.10)	0.06 (192.62)	− 4.05 (− 4.85)	0.06 (0.04)

*Indicates statistically significant signals in algorithm. *ROR* reporting odds ratio; *CI* confidence interval; *PRR* proportional reporting ratio; *χ*^2^, chi-squared; *IC* information component; *IC025* the lower limit of 95% CI of the IC; *EBGM* empirical Bayesian geometric mean; *EBGM05*, the lower limit of 95% CI of EBGM.

### Signal of preferred terms

We further examined PT signals, a total of 97 significant disproportionality PTs conforming to the four algorithms simultaneously (Table 3). Blood and lymphatic system events, gastrointestinal events, cardiovascular events, and nervous system events that are included in the label are usually reported in patients with niraparib. In this study, fatigue (PT: 10016256), nausea (PT: 10028813), vomiting (PT: 10047700), constipation (PT: 10010774), anaemia (PT: 10002034), thrombocytopenia (PT: 10043554), headache (PT: 10019211), hypertension (PT: 10020772), insomnia (PT: 10022437) have been reported in patients treated with niraparib, which are indicated in the label for niraparib. In our analysis, unexpected significant AEs were uncovered in the label, including neuropathy peripheral (PT: 10029331), photosensitivity reaction (PT: 10034972), gastrooesophageal reflux disease (PT: 10017885), emotional distress (PT: 10049119), feeling abnormal (PT: 10016322), blood potassium decreased (PT: 10005724), heart rate increased (PT: 10019303), balance disorder (PT: 10049848), memory impairment (PT: 10027175). However, rash, diarrhea, intestinal perforation, peripheral edema, bronchitis, conjunctivitis, gamma-glutamyl transferase increased, albumin decreased, blood alkaline phosphatase increased, depression and cardiac arrest which were listed in drug label, did not meet the criteria for at least one of the four algorithms.

**Table 3:** Signal strength of reports of niraparib at the preferred term (PT) level in FAERS database.

SOC	Preferred terms (PTs)	Niraparib cases reporting PT	ROR (95% two-sided Cl)	PRR (χ^2^)	IC (IC025)	EBGM (EBGM05)
Blood and lymphatic system disorders	Anaemia	772	8.52 (7.91–9.17)	8.02 (4723.29)	2.97 (2.87)	7.93 (7.37)
	Thrombocytopenia	606	11.02 (10.15–11.97)	10.50 (5148.09)	3.35 (3.22)	10.34 (9.52)
	Lymphadenopathy	127	7.18 (6.02–8.56)	7.11 (660.38)	2.74 (2.48)	7.04 (5.91)
	Pancytopenia	124	4.53 (3.80–5.42)	4.50 (335.57)	2.11 (1.85)	4.47 (3.74)
Cardiac disorders	Palpitations	361	6.21 (5.59–6.90)	6.05 (1514.14)	2.56 (2.41)	6.00 (5.40)
	Tachycardia	121	2.58 (2.15–3.08)	2.56 (115.10)	1.32 (1.06)	2.55 (2.13)
Gastrointestinal disorders	Nausea	3459	11.59 (11.14–12.06)	8.46 (23,269.91)	3.06 (3.00)	8.36 (8.03)
	Constipation	2499	27.71 (26.50–28.99)	22.01 (48,882.17)	4.40 (4.34)	21.29 (20.35)
	Vomiting	1245	5.79 (5.46–6.14)	5.28 (4374.92)	2.39 (2.30)	5.25 (4.95)
	Abdominal pain upper	534	5.14 (4.71–5.61)	4.95 (1686.01)	2.29 (2.16)	4.92 (4.51)
	Abdominal discomfort	525	5.46 (5.00–5.97)	5.26 (1813.06)	2.37 (2.24)	5.23 (4.79)
	Dry mouth	521	14.18 (12.97–15.49)	13.59 (5965.45)	3.70 (3.57)	13.32 (12.19)
	Abdominal pain	396	3.44 (3.11–3.80)	3.36 (659.03)	1.73 (1.58)	3.35 (3.03)
	Dyspepsia	385	8.41 (7.59–9.31)	8.16 (2398.36)	2.98 (2.83)	8.07 (7.29)
	Stomatitis	332	10.06 (9.01–11.23)	9.80 (2591.38)	3.23 (3.07)	9.67 (8.66)
	Abdominal distension	309	5.98 (5.34–6.69)	5.85 (1234.99)	2.51 (2.34)	5.80 (5.18)
	Intestinal obstruction	230	11.51 (10.09–13.13)	11.30 (2124.80)	3.41 (3.21)	11.12 (9.75)
	Flatulence	216	7.74 (6.76–8.86)	7.61 (1228.92)	2.86 (2.66)	7.53 (6.58)
	Gastrooesophageal reflux disease *	175	4.38 (3.77–5.09)	4.33 (446.61)	2.07 (1.85)	4.31 (3.71)
	Dysphagia	169	3.55 (3.05–4.14)	3.52 (303.79)	1.78 (1.55)	3.50 (3.01)
	Ascites	158	10.64 (9.08–12.46)	10.51 (1338.52)	3.28 (3.05)	10.35 (8.83)
	Retching	156	15.28 (13.03–17.93)	15.09 (2005.82)	3.75 (3.52)	14.76 (12.58)
	Oral pain	110	9.36 (7.75–11.31)	9.28 (801.80)	3.08 (2.80)	9.16 (7.58)
General disorders and administration site conditions	Fatigue	3075	9.11 (8.75–9.50)	6.98 (16,200.15)	2.79 (2.73)	6.92 (6.64)
	Asthenia	1158	6.48 (6.10–6.89)	5.94 (4791.56)	2.55 (2.46)	5.89 (5.54)
	Malaise	670	2.69 (2.49–2.91)	2.60 (670.55)	1.37 (1.25)	2.59 (2.40)
	Adverse drug reaction	595	12.40 (11.41–13.48)	11.82 (5807.91)	3.51 (3.39)	11.62 (10.69)
	Disease progression	593	12.00 (11.04–13.04)	11.44 (5574.21)	3.47 (3.34)	11.25 (10.35)
	Feeling abnormal *	557	4.35 (3.99–4.74)	4.19 (1358.61)	2.05 (1.92)	4.17 (3.83)
	Peripheral swelling	259	2.43 (2.15–2.75)	2.40 (213.07)	1.25 (1.07)	2.40 (2.12)
	Drug intolerance	228	3.49 (3.06–3.98)	3.44 (394.47)	1.75 (1.56)	3.43 (3.00)
	Ill-defined disorder	179	6.65 (5.73–7.71)	6.56 (836.56)	2.65 (2.43)	6.50 (5.60)
	Illness	175	5.01 (4.31–5.82)	4.95 (548.93)	2.26 (2.04)	4.92 (4.23)
	Unevaluable event	131	3.08 (2.59–3.66)	3.06 (181.42)	1.58 (1.32)	3.05 (2.57)
	Decreased activity	116	21.91 (18.19–26.39)	21.70 (2214.14)	4.15 (3.88)	21.00 (17.43)
Infections and infestations	Urinary tract infection	340	3.71 (3.33–4.13)	3.63 (648.91)	1.84 (1.68)	3.61 (3.24)
	Nasopharyngitis	238	2.47 (2.17–2.81)	2.44 (202.96)	1.27 (1.08)	2.43 (2.14)
Investigations	Platelet count decreased	2330	54.14 (51.67–56.72)	43.56 (90,943.01)	5.32 (5.26)	40.75 (38.90)
	Blood pressure increased	1346	18.25 (17.24–19.33)	16.27 (18,932.10)	3.97 (3.89)	15.88 (14.99)
	Carbohydrate antigen 125 increased	1331	1067.22 (977.68–1164.95)	945.93 (496,419.82)	8.19 (8.09)	374.25 (342.85)
	Haemoglobin decreased	1051	22.78 (21.36–24.29)	20.82 (19,269.58)	4.31 (4.21)	20.17 (18.92)
	White blood cell count decreased	823	14.70 (13.68–15.79)	13.73 (9554.34)	3.73 (3.62)	13.46 (12.53)
	Red blood cell count decreased	812	61.48 (57.08–66.23)	57.29 (41,149.29)	5.62 (5.52)	52.51 (48.75)
	Heart rate increased	699	15.29 (14.15–16.52)	14.43 (8575.94)	3.79 (3.68)	14.13 (13.08)
	Weight decreased	641	4.39 (4.06–4.76)	4.21 (1578.02)	2.06 (1.94)	4.19 (3.87)
	Blood count abnormal	462	26.7 (24.28–29.35)	25.68 (10,538.43)	4.55 (4.41)	24.7 (22.46)
	Blood creatinine increased	366	11.67 (10.50–12.96)	11.33 (3395.41)	3.44 (3.28)	11.15 (10.04)
	Laboratory test abnormal	304	14.94 (13.31–16.76)	14.58 (3762.72)	3.77 (3.60)	14.26 (12.71)
	Haematocrit decreased	262	30.32 (26.75–34.37)	29.67 (6930.65)	4.68 (4.49)	28.35 (25.01)
	Full blood count decreased	229	19.69 (17.24–22.49)	19.32 (3862.25)	4.12 (3.92)	18.77 (16.43)
	Neutrophil count decreased	185	9.13 (7.89–10.57)	9.00 (1299.47)	3.08 (2.87)	8.89 (7.68)
	Tumour marker increased	183	62.72 (53.82–73.10)	61.76 (9947.24)	5.43 (5.20)	56.24 (48.25)
	Blood potassium decreased *	167	11.20 (9.60–13.07)	11.06 (1502.89)	3.35 (3.13)	10.88 (9.33)
	Blood magnesium decreased	158	35.06 (29.84–41.20)	34.60 (4884.26)	4.76 (4.53)	32.82 (27.93)
	Eastern Cooperative Oncology Group performance status worsened	133	248.74 (203.29–304.36)	245.93 (23,204.79)	6.24 (5.96)	176.17 (143.98)
	Computerised tomogram abnormal	101	145.98 (117.45–181.43)	144.73 (11,680)	5.76 (5.45)	117.44 (94.49)
Metabolism and nutrition disorders	Decreased appetite	1176	10.19 (9.59–10.83)	9.27 (8639.33)	3.18 (3.09)	9.14 (8.61)
	Dehydration	393	6.33 (5.73–7.01)	6.16 (1689.59)	2.59 (2.44)	6.1 (5.52)
Musculoskeletal and connective tissue disorders	Arthralgia	678	3.31 (3.07–3.58)	3.18 (1026.84)	1.66 (1.54)	3.17 (2.93)
	Back pain	490	4.09 (3.74–4.48)	3.96 (1089.14)	1.97 (1.83)	3.94 (3.60)
	Pain in extremity	391	2.57 (2.32–2.84)	2.52 (360.79)	1.32 (1.17)	2.51 (2.27)
	Myalgia	292	3.39 (3.02–3.81)	3.33 (477.10)	1.71 (1.54)	3.32 (2.95)
	Muscle spasms	276	2.90 (2.58–3.27)	2.86 (334.64)	1.50 (1.32)	2.85 (2.53)
	Muscular weakness	167	2.98 (2.56–3.48)	2.96 (216.03)	1.53 (1.31)	2.95 (2.53)
	Bone pain	160	5.08 (4.34–5.94)	5.02 (512.91)	2.28 (2.04)	4.99 (4.27)
	Arthritis	122	2.96 (2.48–3.54)	2.94 (156.24)	1.52 (1.25)	2.93 (2.45)
Nervous system disorders	Headache	1318	4.19 (3.96–4.44)	3.83 (2823.61)	1.93 (1.84)	3.81 (3.60)
	Dizziness	905	3.75 (3.50–4.01)	3.54 (1673.98)	1.81 (1.71)	3.52 (3.29)
	Neuropathy peripheral *	649	12.93 (11.94–14.01)	12.27 (6616.89)	3.56 (3.45)	12.05 (11.12)
	Hypoaesthesia	235	3.16 (2.77–3.59)	3.11 (337.60)	1.61 (1.42)	3.10 (2.73)
	Balance disorder *	220	4.83 (4.23–5.53)	4.76 (651.15)	2.21 (2.01)	4.73 (4.14)
	Tremor	215	2.54 (2.22–2.91)	2.51 (196.32)	1.31 (1.11)	2.51 (2.19)
	Dysgeusia	213	7.00 (6.11–8.02)	6.89 (1063.97)	2.73 (2.53)	6.83 (5.96)
	Memory impairment *	205	2.58 (2.25–2.96)	2.55 (193.94)	1.33 (1.13)	2.55 (2.22)
	Taste disorder	174	16.26 (13.97–18.92)	16.03 (2392.61)	3.84 (3.62)	15.65 (13.45)
Psychiatric disorders	Insomnia	1722	14.50 (13.77–15.27)	12.51 (18,093.9)	3.61 (3.53)	12.28 (11.67)
	Anxiety	554	3.59 (3.30–3.91)	3.47 (981.34)	1.78 (1.65)	3.45 (3.17)
	Emotional distress *	429	5.15 (4.68–5.68)	5.00 (1372.11)	2.30 (2.15)	4.97 (4.51)
	Sleep disorder	330	8.94 (8.01–9.98)	8.72 (2230.66)	3.07 (2.91)	8.61 (7.71)
	Stress	171	4.56 (3.92–5.31)	4.51 (465.39)	2.13 (1.90)	4.49 (3.85)
	Nervousness	125	4.89 (4.10–5.84)	4.85 (379.83)	2.21 (1.95)	4.82 (4.04)
	Middle insomnia	110	13.52 (11.18–16.35)	13.40 (1236.51)	3.55 (3.27)	13.14 (10.87)
Renal and urinary disorders	Renal impairment	320	6.65 (5.95–7.44)	6.50 (1479.45)	2.66 (2.49)	6.44 (5.76)
	Renal disorder	100	3.78 (3.10–4.60)	3.75 (201.25)	1.85 (1.56)	3.74 (3.07)
Respiratory, thoracic and mediastinal disorders	Dyspnoea	859	2.92 (2.73–3.13)	2.78 (1001.66)	1.47 (1.36)	2.77 (2.59)
	Cough	425	2.85 (2.58–3.14)	2.78 (488.14)	1.46 (1.32)	2.77 (2.51)
	Oropharyngeal pain	206	4.26 (3.71–4.89)	4.20 (501.03)	2.03 (1.83)	4.18 (3.64)
	Dyspnoea exertional	160	7.15 (6.11–8.37)	7.07 (825.90)	2.75 (2.51)	7.00 (5.98)
	Epistaxis	153	3.57 (3.04–4.19)	3.54 (277.98)	1.78 (1.55)	3.52 (3.00)
	Rhinorrhoea	149	4.09 (3.48–4.81)	4.05 (340.73)	1.97 (1.73)	4.03 (3.42)
	Pleural effusion	113	3.61 (3.00–4.34)	3.58 (209.74)	1.79 (1.52)	3.57 (2.96)
Skin and subcutaneous tissue disorders	Photosensitivity reaction *	204	23.58 (20.48–27.15)	23.19 (4177.97)	4.33 (4.13)	22.39 (19.44)
	Hyperhidrosis	167	2.59 (2.22–3.02)	2.57 (160.02)	1.33 (1.11)	2.56 (2.20)
Vascular disorders	Hypertension	715	7.14 (6.62–7.71)	6.77 (3509.00)	2.73 (2.62)	6.71 (6.21)
	Hot flush	219	5.98 (5.23–6.84)	5.89 (882.89)	2.51 (2.31)	5.84 (5.11)
	Blood pressure fluctuation	164	9.74 (8.34–11.38)	9.62 (1248.94)	3.16 (2.94)	9.49 (8.12)
	Thrombosis	112	2.61 (2.17–3.15)	2.60 (109.78)	1.34 (1.06)	2.59 (2.15)

*Emerging findings of niraparib associated AEs from FAERS database. *ROR* reporting odds ratio; *CI* confidence interval; *PRR* proportional reporting ratio; *χ* chi-squared; *IC* information component; *IC025* the lower limit of 95% CI of the IC; *EBGM* empirical Bayesian geometric mean; *EBGM05* the lower limit of 95% CI of EBGM.

### Onset time of events

**Figure 1:**
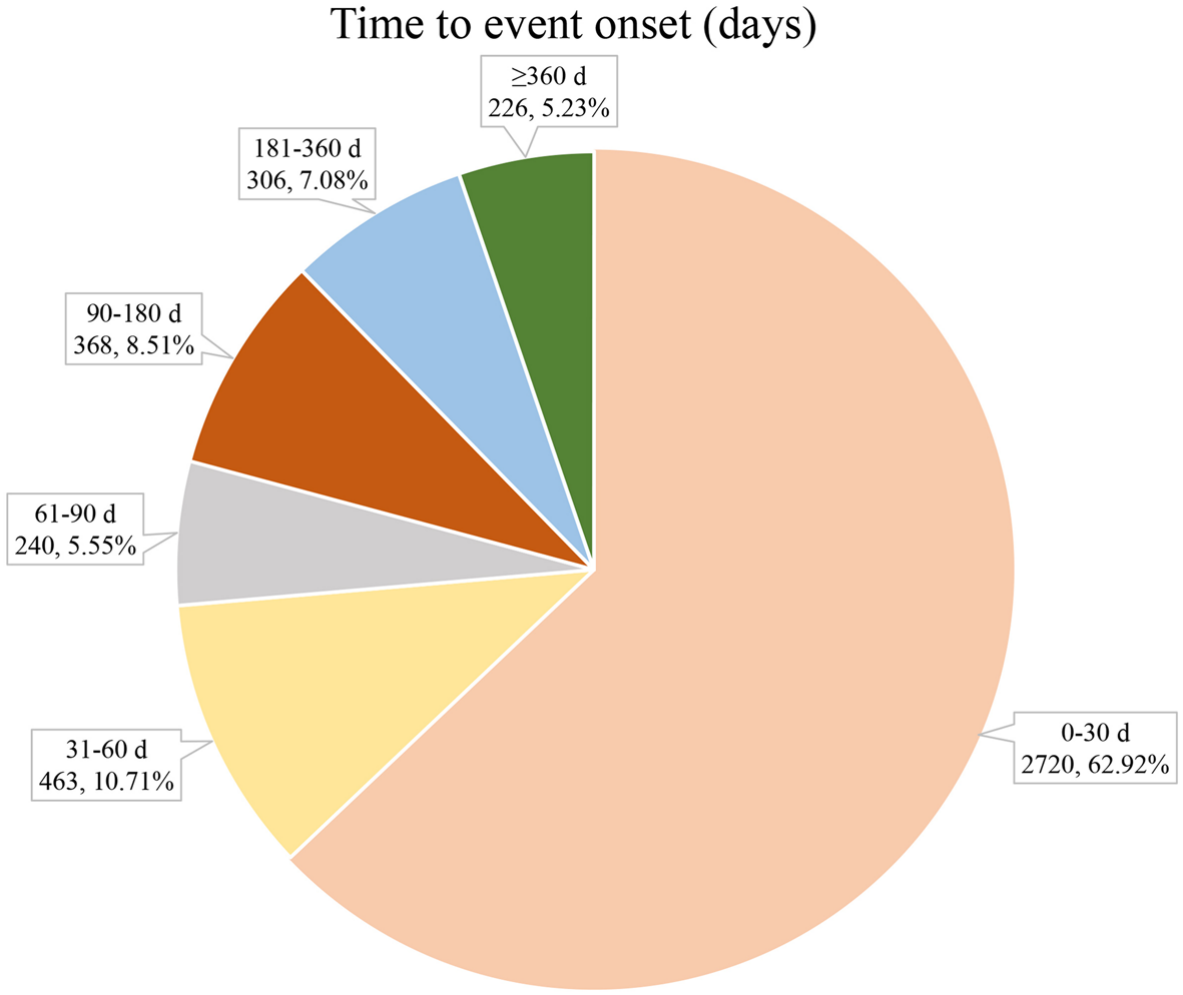
Time to onset of niraparib-related AEs.

The onset times of niraparib-associated AEs were collected from the database. Excluding unreported or unknown onset time reports, a total of 4323 AEs (36.95%, data available in 4323/11,701) reported onset time and the median onset time was 18 days (interquartile range [IQR] 14–48 days). As shown in Fig. 1, results indicated that the onsets of niraparib were variable, most of the cases occurred within the first 1 (n = 2720, 62.92%), 2 (n = 10.71%) and 3 months (n = 240, 5.55%) after niraparib initiation. Notably, AEs might still occur after 1 year of niraparib treatment with percentage of 5.22% as illustrated in our data.

## Discussion

This large real-world comparison of niraparib leveraged FAERS data and demonstrated that the most commonly reported and significant signals at SOC levels, likelihood of gastrointestinal disorders, blood and lymphatic system disorders, general disorders, administration site conditions, investigations, nervous system disorders, etc. In contrast, significant disproportionality of AEs in the skin and subcutaneous tissue disorders, renal and urinary disorders, cardiac disorders, infections and infestations are less common. It's worth noting that, we did not find disproportionality reporting for endocrine systems, such as adrenal insufficiency, thyroid dysfunction, and hypophysitis.

The AEs of niraparib occurred more commonly in females (78.55%) than in males (1.79%), due to the specific indications for ovarian and fallopian tube cancer. PARP inhibitors (PARPi) such as niraparib have been approved for different malignancies with genomic alteration in germline breast cancer susceptibility gene (BRCA) and DNA damage response (DDR) pathway genes. Studies have demonstrated that PARP inhibitor efficacy in BRCA mutated ovarian and breast cancer, prostate cancer, pancreatic cancer and small cell lung carcinoma (SCLC)^14^. In addition to the label indications, niraparib has great potential for the treatment of cholangiocarcinoma tumor^15^, prostate tumor^16,17^, breast cancer^18,19^ and lung cancer^20^. This study also illustrated a higher AEs proportion in elderly patients (33.54% > 50 years), which was consistent with the epidemiology of ovarian cancer. The risk of ovarian cancer increases in females with age^21^, although there was an overall decreasing trend of incidence and mortality of ovarian cancer over the past decade, a substantial increase in incidence was observed in younger females in some countries^22^. Furthermore, physicians may need to exercise additional caution when using niraparib in the elderly population, given the complex/various comorbidities that these patients typically experience.

The signal of niraparib in tumour marker increased, carbohydrate antigen 125 increased may be due to the failure of one prior systemic therapy, and patients may still have tumor metastasis and tumor progression when the efficacy of niraparib is imperfect. Moreover, owing to the characteristics of local invasion and distant metastasis of malignant tumors, it might be illogical to judge whether tumor metastasis and tumor progression are caused by niraparib only by ADR signals^23^.

Based on the disproportionality analysis, many organs or tissues can be involved, although some AEs reported much more commonly than others. Results demonstrated that the most commonly significant signals at SOC levels were general disorders, administration site conditions, investigations, gastrointestinal disorders, nervous system disorders, injury, poisoning and procedural complications. Among them, significant AEs mainly included anaemia, dizziness, abdominal pain, constipation, nausea, anemia, blood magnesium decreased and blood pressure increased which were corresponding to that in the instruction and clinical safety data^4,8,24^.

The signals of disproportionality reporting in FAERS showed a high risk in blood and lymphatic system. Findings from postmarketing studies suggest the risk may be signifcant. Studies have shown that haematological events were the most commonly occurring grade ≥ 3 AEs in patients with ovarian cancer for niraparib treatment^11^. Grade ≥ 3 AEs with an incidence ≥ 10% in niraparib recipients included anaemia (31.0% of niraparib recipients and 1.6% of placebo recipients), thrombocytopenia (28.7% and 0.4%), platelet count decreased (13.0% and 0%) and neutropenia (12.8% and 1.2%). The majority of niraparib treatment discontinuations were due to myelosuppressive events, including thrombocytopenia (4.3%), leukopenia (2.1%), anaemia (1.9%) and neutropenia (1.9%)^6^. Our results are in agreement with the studies regarding the disproportionately high reporting found for blood and lymphatic system disorders. Myelosuppression were the main reason for discontinuation but were relatively infrequent , and the niraparib package insert advises checking complete blood counts periodically. There is a growing concern that patients treated with niraparib may experience an increased risk in blood and lymphatic system. Haematological reactions were managed with monitoring and dose reduction or interruption. Careful use of antiplatelet and anti-coagulants is recommended in patients receiving niraparib due to its increased risk of thrombocytopenia. It is important to note that niraparib does not currently have a ‘boxed warning’ for an increased risk of myelosuppression. Given the observed increased reporting risk for anemia, thrombocytopenia as well as neutropenia with niraparib found in the current study, additional analyses are warranted.

For cardiovascular events, such as hypertension, palpitations and tachycardia, the underlying mechanism of niraparib-induced cardiotoxicity remains unclear. Based on investigations in preclinical models, PARP inhibition appears to be limit or inhibit cardiovascular dysfunction^25^. According to the specification of ZEJULA (niraparib) capsules, niraparib caused effects on pulse rate and blood pressure, which may be related to pharmacological inhibition of the dopamine, norepinephrine, and serotonin transporter^3^. Besides, the UK's Medicines and Healthcare Products Regulatory Agency (MHRA) recently issued a warning about the risk of severe hypertension and posterior reversible encephalopathy syndrome in niraparib.

Gastrointestinal events are frequent during niraparib treatment, including abdominal pain, nausea, constipation, and vomiting, of which nausea is the most common event. As we all know, gastrointestinal toxicity is a well-known side effect of PARP inhibitors which is mediated via off-target kinase inhibition. These types of AEs (e.g., nausea) are common for kinase inhibitors and often occurring early after treatment initiation. Patients should be prescribed antiemetics (e.g., ondansetron or prochlorperazine) to take as needed. A trial of scheduled antiemetics prior to each dose can be considered in patients with persistent emesis, while dose reductions can be considered in refractory cases.

It's important to note that the neurological AEs associated with niraparib, which the most common is insomnia. A study in Sweden showed that PARP1- and CTCF-regulated contacts between circadian loci and the repressive chromatin environment at the lamina therefore mediate circadian transcriptional plasticity^26^. The neurological AEs should be distinguished from other causes, such as central nervous system involvement and endocrine abnormalities.

It's worth noting that we found disproportionality reporting for the psychiatric disorders, such as emotional distress, anxiety, stress, nervousness et al. About 50% of cancer patients have in fact been shown to have psychiatric disorders as a consequence of cancer at some point during the cancer trajectory^27^. Mood disorders are common in patients with female genital malignancy, so appropriate psychological intervention is necessary.

Unexpected and significant safety signals such as neuropathy peripheral, photosensitivity reaction, gastrooesophageal reflux disease, blood potassium decreased, balance disorder, memory impairmentand, emotional distress were detected in our analysis. A meta-analysis shows that PARP inhibition does not appear to reduce the risk of chemotherapy-induced peripheral neuropathy^28^. Whether niraparib may lead to neuropathy remains an area in need of further investigation. The decrease in blood potassium may be associated with vomiting. At present, the mechanism of niraparib-induced balance disorder and memory impairment are not completely understood. The possible potential mechanism may be the unique pharmacology of niraparib including better blood–brain-barrier penetrance over other PARP inhibitors, which has been shown to induce intracranial response in animal models^29^. In addition, we did not find relevant literature reports about other significant new signals, like photosensitivity reactions, gastrooesophageal reflux, and emotional distress. Accordingly, further clinical studies are necessary to understand the pathogenesis of these adverse events.

Although the niraparib package insert and clinical case reports have indicated that it can cause rash, diarrhea, intestinal perforation, peripheral edema, bronchitis, conjunctivitis, gamma-glutamyl transferase increased, albumin decreased, blood alkaline phosphatase increased, depression and cardiac arrest in ovarian cancer cancer patients, the signal strength did not meet the criteria in our analysis.

Results of this study indicated that the median onset time was 18 days, and most of the cases occurred within the first 1 (n = 2720, 62.92%), 2 (n = 10.71%) and 3 months (n = 240, 5.55%) after niraparib. Most of the AEs occurred with in the first three months. Besides, the ENGOT-OV16/NOVA trial in the gBRCAmut and non-gBRCAmut cohorts, niraparib treatment resulted in a mean time with toxicity of 0.28 years and 0.10 years, respectively^30^. These results suggested that we should be vigilant about the AEs associated with niraparib in the first month and early recognition of AEs caused by niraparib therapy is important due to these adverse drug effects can be life-threatening.

Although the current study showed a potentially insightful relationship between the use of niraparib and the odds of reporting AEs in the FAERS, it is not without limitations. In essence, voluntary reports are not restricted to health care professionals, and consumers are also candidates to report AEs. Whereas the medical expertise of the consumer is limited, unfortunately. As the FDA does not require a proof of causal relationship for submitted reports^31^, partial correlate AEs maybe open to debate. Due to the lack of information in the FAERS database, it is difficult to control confounding factors such as age, comorbidities or other factors that may have an impact on health. Furthermore, with all the advantages brought by the data mining techniques conducted in this study, it should not be overlooked that this method does not address every problem of detecting and analyzing adverse drug reaction signals based on spontaneous reporting systems. Although data mining techniques cannot compensate for the inherent limitations of a spontaneous reporting system and substitute for expert review, it does have a place particularly where large volumes of data are involved^32^. It is reassuring, however, that all signals identified in the previous system that went on to become frequently reported in the WHO database were also identified in the retrospective BCPNN analysis^33^.

## Conclusion

By analysis of FAERS data comprehensively and systematically, we identified risk tendencies and time to AEs onsets with niraparib. Unexpected and new significant AEs as neuropathy peripheral, photosensitivity reaction and gastrooesophageal reflux disease might also occur. Common haemal, cardiovascular, and gastrointestinal AEs should be highly concerned. Clinicians should be aware of severe adverse reactions to tailor their agent choice and monitor their patients accordingly. We found multiple postmarketing safety signals which were similar to the clinical trials, as well as other reports that required further regulatory investigation to determine their significance. More research is needed in the future to explain the safety of niraparib for better application.

## Materials and methods

### Data source and processing

The FDA publishes FAERS files every quarter (i.e., four files each year). In our study, AE reports linked to niraparib submitted in the FAERS database from Q1 2017 (first approval period) to Q4 2021 (latest FAERS update) were extracted. Since the database is updated quarterly, it will inevitably duplicate the previous public reports, and the deduplication process is performed before statistical analysis in our study. After processing the original FAERS dataset, we created a dataset caused by the reports of niraparib.

### Adverse events and drug detection

AEs in FAERS reports are coded using the Medical Dictionary for Regulatory Activities (MedDRA) of Preferred Terms (PTs). All individual AEs reports of niraparib recorded in system organ class (SOC) and PT levels were identified to describe the toxicity spectrum. The drugs in the FAERS database can be arbitrarily reported. Code for drug’s reported role in event include primary suspect drug (PS), secondary suspect drug (SS), concomitant (C), and interacting (I). As a result, our target drugs were defined as generic name (niraparib) and trade name (Zejula) in the DRUG file, and choose the role_cod as PS.

### Data mining algorithm

We performed a disproportionality analysis to identify potential associations between niraparib and AEs. The disproportionality analysis was considered to be an important analytical tool in pharmacovigilance, and four statistical procedures were applied: reporting odds ratio (ROR), the proportional reporting ratio (PRR), the Bayesian confidence propagation neural network (BCPNN), and the multi-item gamma Poisson shrinker (MGPS)^34^. By using these methods, we can identify the drug-events^35^, as well as compare the proportion of adverse events occurring between a specific study drug and all other drugs. Four algorithms were applied to quantify the signals of niraparib-associated AEs in our study, and the equations and criteria were described as previously and shown in Supplementary Table S2. These algorithms’ extraction decision rules were used to detect signals and calculate scores to measure associations between drugs and AEs. When a methodological threshold was met, the signals could be detected in SOC level. Basically, the higher the score of the four parameters, the stronger disproportion appears to be. In our study, we also discovered AE signals that simultaneously met the four algorithm standards in PT level^36,37^. The novelty/unexpectedness signals were defined as significant AEs which were not listed in the instructions/product label.

The time-to-onset of AEs were calculated by subtracting the start date of niraparib from the onset date of report. We removed the reports with input errors (EVENT_DT earlier than START_DT) and the inaccurate date entries. The median and interquartile ranges were used to describe the time-to-onset. All data processing and statistical analyses were performed using MYSQL 8.0, Navicat Premium 15, Microsoft EXCEL 2019 and the GraphPad Prism 8 (GraphPad Software, CA, USA).

### Ethical approval

This article does not contain any studies with human participants or animals performed by any of the authors.

## Supplementary Information

Supplementary Information.

## Data Availability

The datasets generated during and/or analyzed during the current study are available from the corresponding author on reasonable request.
