# Supplementary material for: A real-world pharmacovigilance study of FDA adverse event reporting system (FAERS) events for niraparib
Source: Sci Rep. 2022 Nov 29;12:20601. doi: 10.1038/s41598-022-23726-4 (PMC9709073; doi:10.1038/s41598-022-23726-4)
Supplement: Supplementary file 1 — Supplementary Information. [file 41598_2022_23726_MOESM1_ESM.docx]

**A real-world** **pharmacovigilance study of FDA Adverse Event Reporting System (FAERS) events for Niraparib**

Menglin Guo^1,+^, Yamin Shu^1,+^, Guosong Chen^1^, Juan Li^1,^* & Feie Li^1,^*

^1^ Department of Pharmacy, Tongji Hospital, Tongji Medical College of Huazhong University of Science and Technology, Wuhan, China

| SOC | Preferred Terms (PTs) | Niraparib Cases Reporting PT | ROR (95% two-sided Cl) | PRR (χ^2^) | IC (IC025) | EBGM (EBGM05) |
| --- | --- | --- | --- | --- | --- | --- |
| Blood and lymphatic system disorders | Thrombocytopenia | 222 | 1604.09 (1536.79-1674.34) | 117.23 (1811444.35) | 5.88 (5.86) | 59.11 (56.63) |
|  | Anaemia | 169 | 1559.26 (1495.73-1625.49) | 84.21 (1842452.54) | 5.41 (5.39) | 42.6 (40.87) |
|  | Pancytopenia | 52 | 1510.90 (1444.95-1579.87) | 221.43 (1674540.65) | 6.79 (6.75) | 111.22 (106.36) |
|  | Lymphadenopathy | 12 | 1494.51 (1419.48-1573.51) | 433.33 (1396269.81) | 7.71(7.66) | 217.17 (206.26) |
| Cardiac disorders | Palpitations | 30 | 1497.27 (1432.7-1564.74) | 207.39 (1689793.84) | 6.69 (6.66) | 104.19 (99.7) |
|  | Tachycardia | 26 | 1491.42 (1428.86-1556.72) | 166.29 (1740686.74) | 6.38 (6.35) | 83.65 (80.14) |
| Gastrointestinal disorders | Nausea | 385 | 1632.06 (1564.95-1702.04) | 28.78 (1869053.09) | 3.9 (3.88) | 14.89 (14.28) |
|  | Constipation | 214 | 1596.52 (1529.99-1665.94) | 108.14 (1820564.31) | 5.77 (5.74) | 54.57 (52.3) |
|  | Vomiting | 164 | 1526.90 (1466.15-1590.16) | 46.83 (1869765.54) | 4.58 (4.56) | 23.91 (22.96) |
|  | Abdominal pain | 57 | 1493.12 (1433.31-1555.42) | 87.67 (1834090.48) | 5.47 (5.45) | 44.34 (42.56) |
|  | Dry mouth | 54 | 1516.86 (1445.16-1592.12) | 341 (1523432.25) | 7.39 (7.34) | 171 (162.92) |
|  | Abdominal pain upper | 49 | 1499.67 (1438-1563.98) | 130.35 (1785864.36) | 6.0 3(6.01) | 65.68 (62.98) |
|  | Intestinal obstruction | 45 | 1514.07 (1435.8-1596.6) | 471.75 (1354007.11) | 7.83 (7.77) | 236.37 (224.15) |
|  | Abdominal discomfort | 42 | 1495.67 (1434.22-1559.74) | 130.55 (1785169.38) | 6.04 (6.01) | 65.77 (63.07) |
|  | Dyspepsia | 34 | 1502.77 (1435.34-1573.36) | 266.43 (1615461.51) | 7.05 (7.01) | 133.72 (127.72) |
|  | Abdominal distension | 33 | 1500.70 (1434.75-1569.67) | 235.2 (1655010.38) | 6.87 (6.83) | 118.1 (112.91) |
|  | Ascites | 32 | 1505.48 (1431.26-1583.55) | 405.41 (1436675.68) | 7.63 (7.57) | 203.2 (193.19) |
|  | Stomatitis | 31 | 1500.74 (1433.74-1570.87) | 259.52 (1623863.38) | 7.01 (6.97) | 130.26 (124.45) |
|  | Dysphagia | 18 | 1490.64 (1426.33-1557.86) | 210.67 (1684397.6) | 6.72 (6.68) | 105.83 (101.27) |
|  | Flatulence | 17 | 1497.38 (1422.11-1576.64) | 434.72 (1395546.83) | 7.72 (7.67) | 217.86 (206.91) |
|  | Gastrooesophageal reflux disease | 17 | 1493.29 (1426.15-1563.59) | 272.59 (1605284.59) | 7.08 (7.04) | 136.79 (130.64) |
|  | Retching | 16 | 1499.90 (1400.4-1606.48) | 789.34 (932548.17) | 8.4 (8.32) | 395.17 (368.96) |
|  | Oral pain | 9 | 1495.29 (1405.08-1591.29) | 677 (1077017.96) | 8.26 (8.19) | 339 (318.55) |
| General disorders and administration site conditions | Fatigue | 283 | 1565.94 (1502.83-1631.69) | 30 (1868841.3) | 3.95 (3.94) | 15.5 (14.88) |
|  | Disease progression | 275 | 1645.37 (1574.54-1719.4) | 145.05 (1784945.56) | 6.19 (6.16) | 73.03 (69.88) |
|  | Asthenia | 118 | 1516.64 (1456.15-1579.64) | 64.38 (1858016.71) | 5.03 (5.01) | 32.69 (31.39) |
|  | Malaise | 98 | 1499.63 (1440.27-1561.44) | 58.06 (1862048.95) | 4.88 (4.87) | 29.53 (28.36) |
|  | Feeling abnormal | 54 | 1503.82 (1441.63-1568.69) | 137.86 (1777318.63) | 6.11 (6.09) | 69.43 (66.56) |
|  | Adverse drug reaction | 42 | 1506.18 (1439.68-1575.75) | 240.63 (1649222.61) | 6.9 (6.87) | 120.81 (115.48) |
|  | Drug intolerance | 35 | 1492.28 (1430.89-1556.29) | 134.22 (1780343.57) | 6.08 (6.05) | 67.61 (64.83) |
|  | Peripheral swelling | 19 | 1479.91 (1419.87-1542.49) | 116.9 (1799748.17) | 5.88 (5.85) | 58.95 (56.56) |
|  | Unevaluable event | 11 | 1489.76 (1422.96-1559.7) | 269.53 (1608397.34) | 7.06 (7.02) | 135.27 (129.2) |
|  | Illness | 9 | 1492.35 (1418.86-1569.65) | 406.33 (1430800.58) | 7.63 (7.58) | 203.67 (193.64) |
|  | Decreased activity | 7 | 1495.55 (1374.38-1627.39) | 988.37 (667736.16) | 8.5 (8.39) | 494.68 (454.61) |
|  | Ill-defined disorder | 6 | 1490.72 (1417.13-1568.13) | 410.11 (1425263.23) | 7.64 (7.59) | 205.56 (195.41) |
| Infections and infestations | Urinary tract infection | 36 | 1490.27 (1429.51-1553.61) | 120.27 (1796852.91) | 5.92 (5.89) | 60.63 (58.16) |
|  | Nasopharyngitis | 24 | 1485.07 (1424.3-1548.43) | 128.77 (1786090) | 6.02 (5.99) | 64.89 (62.23) |
| Investigations | Platelet count decreased | 359 | 1709.4 (1633.64-1788.68) | 170.38 (1764267.47) | 6.42 (6.38) | 85.69 (81.89) |
|  | Carbohydrate antigen 125 increased | 140 | 1575.85 (1375.84-1804.94) | 1333.44 (302972.85) | 8.08 (7.91) | 667.22 (582.53) |
|  | Haemoglobin decreased | 111 | 1543.34 (1477.01-1612.65) | 184.69 (1725760.98) | 6.53 (6.5) | 92.84 (88.85) |
|  | Blood pressure increased | 105 | 1535.84 (1471.33-1603.19) | 150.01 (1766646.03) | 6.23 (6.2) | 75.51 (72.33) |
|  | White blood cell count decreased | 79 | 1520.63 (1456.9-1587.15) | 152.73 (1761437.73) | 6.26 (6.23) | 76.86 (73.64) |
|  | Heart rate increased | 78 | 1528.13 (1459.62-1599.85) | 257.07 (1633061.73) | 7 (6.96) | 129.04 (123.25) |
|  | Red blood cell count decreased | 71 | 1530.03 (1447.75-1617) | 525.37 (1291763.4) | 7.96 (7.9) | 263.18 (249.03) |
|  | Blood creatinine increased | 65 | 1519.18 (1452.27-1589.18) | 232.57 (1662083.14) | 6.86 (6.82) | 116.79 (111.64) |
|  | Weight decreased | 58 | 1493.76 (1433.91-1556.1) | 87.83 (1833970.1) | 5.47 (5.45) | 44.42 (42.64) |
|  | Neutrophil count decreased | 47 | 1511.9 (1441.94-1585.26) | 310.53 (1561130.94) | 7.26 (7.22) | 155.77 (148.56) |
|  | Haematocrit decreased | 37 | 1511.03 (1423.18-1604.31) | 630.95 (1146169.84) | 8.18 (8.11) | 315.97 (297.6) |
|  | Laboratory test abnormal | 22 | 1498.56 (1427.53-1573.11) | 349.51 (1506972.01) | 7.42 (7.38) | 175.25 (166.95) |
|  | Tumour marker increased | 22 | 1504.4 (1365.93-1656.91) | 1095.64 (535054.76) | 8.46 (8.33) | 548.32 (497.85) |
|  | Full blood count decreased | 21 | 1500.52 (1421.66-1583.76) | 496.49 (1316174.84) | 7.89 (7.83) | 248.75 (235.67) |
|  | Blood magnesium decreased | 17 | 1501.15 (1383.63-1628.65) | 959.46 (710491.19) | 8.5 (8.4) | 480.23 (442.63) |
|  | Blood potassium decreased | 16 | 1498.28 (1416.2-1585.13) | 553.25 (1240940.93) | 8.03 (7.97) | 277.13 (261.94) |
|  | Eastern Cooperative Oncology Group performance status worsened | 13 | 1499.78 (1266.02-1776.69) | 1354.54 (190734.82) | 7.64 (7.42) | 677.77 (572.13) |
|  | Computerised tomogram abnormal | 8 | 1496.95 (1252.77-1788.72) | 1365.33 (173165.84) | 7.53 (7.3) | 683.17 (571.73) |
| Metabolism and nutrition disorders | Decreased appetite | 123 | 1534.29 (1471.93-1599.29) | 93.15 (1831476.37) | 5.56 (5.53) | 47.08 (45.16) |
|  | Dehydration | 56 | 1508.06 (1444.76-1574.13) | 160.45 (1750311.77) | 6.33 (6.3) | 80.73 (77.34) |
| Musculoskeletal and connective tissue disorders | Arthralgia | 64 | 1480.93 (1422.61-1541.63) | 59.3 (1860144.02) | 4.91 (4.9) | 30.15 (28.96) |
|  | Back pain | 49 | 1493.12 (1432.86-1555.91) | 101.4 (1819008.44) | 5.68 (5.65) | 51.2 (49.14) |
|  | Pain in extremity | 34 | 1480.11 (1421.04-1541.64) | 88.08 (1832634.5) | 5.48 (5.45) | 44.54 (42.76) |
|  | Muscle spasms | 31 | 1490.19 (1428.88-1554.12) | 135.41 (1778647.3) | 6.09 (6.06) | 68.21 (65.4) |
|  | Myalgia | 28 | 1487.54 (1426.59-1551.09) | 129.85 (1785068.52) | 6.03 (6) | 65.43 (62.75) |
|  | Muscular weakness | 14 | 1488.03 (1424.07-1554.86) | 205.66 (1690306.15) | 6.68 (6.65) | 103.33 (98.89) |
|  | Bone pain | 13 | 1492.74 (1423.3-1565.57) | 323.55 (1539000.88) | 7.32 (7.27) | 162.28( 154.73) |
|  | Arthritis | 7 | 1488.37 (1420.62-1559.36) | 292.65 (1578025.96) | 7.18 (7.14) | 146.83 (140.14) |
| Nervous system disorders | Headache | 119 | 1486.46 (1428.11-1547.19) | 38.79 (1870058.22) | 4.31 (4.3) | 19.89 (19.11) |
|  | Dizziness | 92 | 1492.49 (1433.62-1553.78) | 54.25 (1864329.78) | 4.79 (4.77) | 27.62 (26.54) |
|  | Neuropathy peripheral | 66 | 1514.8 (1450.79-1581.62) | 168.35 (1741538.55) | 6.4 (6.37) | 84.68 (81.1) |
|  | Dysgeusia | 24 | 1500.41 (1427.72-1576.8) | 381.12 (1466482.54) | 7.54 (7.49) | 191.06 (181.8) |
|  | Balance disorder | 20 | 1495.64 (1427.6-1566.91) | 289.39 (1584132.1) | 7.16 (7.12) | 145.19 (138.59) |
|  | Hypoaesthesia | 19 | 1488.34 (1425.6-1553.83) | 175.07 (1729206.81) | 6.45 (6.42) | 88.03 (84.32) |
|  | Memory impairment | 18 | 1490.81 (1426.38-1558.15) | 213.15 (1681255.77) | 6.73 (6.7) | 107.07 (102.45) |
|  | Tremor | 14 | 1482.76 (1421.3-1546.87) | 150.63 (1758994.06) | 6.24 (6.21) | 75.81 (72.67) |
|  | Taste disorder | 8 | 1494.9 (1402.55-1593.33) | 705.79 (1038944.63) | 8.3 (8.23) | 353.4 (331.56) |
| Psychiatric disorders | Insomnia | 145 | 1553.49 (1489.35-1620.4) | 112.37 (1812190.07) | 5.82 (5.8) | 56.69 (54.34) |
|  | Emotional distress | 58 | 1499.1 (1438.4-1562.36) | 104.17 (1816421.99) | 5.71 (5.69) | 52.58 (50.46) |
|  | Anxiety | 47 | 1486.27 (1426.94-1548.07) | 84.93 (1836471.09) | 5.42 (5.4) | 42.96 (41.25) |
|  | Sleep disorder | 27 | 1500.41 (1431.02-1573.16) | 312.61 (1555340.56) | 7.27 (7.23) | 156.81 (149.55) |
|  | Stress | 19 | 1497.12 (1425.62-1572.2) | 361.06 (1491505.46) | 7.47 (7.42) | 181.03 (172.38) |
|  | Middle insomnia | 11 | 1497.29 (1392.96-1609.44) | 842.59 (860813.42) | 8.45 (8.36) | 421.79 (392.4) |
|  | Nervousness | 6 | 1492.29 (1412.36-1576.75) | 523.27 (1277415.52) | 7.96 (7.9) | 262.13 (248.09) |
| Renal and urinary disorders | Renal impairment | 96 | 1532.61 (1467.58-1600.53) | 168.09 (1744401.17) | 6.4 (6.36) | 84.55 (80.96) |
|  | Renal disorder | 5 | 1490.21 (1416.49-1567.78) | 413.35 (1420832.4) | 7.65 (7.6) | 207.17 (196.93) |
| Respiratory, thoracic and mediastinal disorders | Dyspnoea | 103 | 1477.08 (1419.26-1537.24) | 38.78 (1869732.18) | 4.31 (4.3) | 19.89 (19.11) |
|  | Cough | 54 | 1490.35 (1430.79-1552.4) | 85.08 (1836617.92) | 5.43( 5.4) | 43.04 (41.32) |
|  | Oropharyngeal pain | 22 | 1494.77 (1428.87-1563.7) | 242.25 (1644733.68) | 6.91 (6.88) | 121.63 (116.26) |
|  | Epistaxis | 20 | 1494.05 (1427.82-1563.35) | 250.84 (1633528.9) | 6.96 (6.92) | 125.92 (120.34) |
|  | Pleural effusion | 18 | 1492.48 (1426.72-1561.26) | 242 (1644571.56) | 6.91 (6.87) | 121.5 (116.15) |
|  | Rhinorrhoea | 18 | 1495.01 (1426.31-1567.02) | 304.71 (1564110.99) | 7.23 (7.19) | 152.86 (145.83) |
|  | Dyspnoea exertional | 14 | 1494.01 (1423.22-1568.33) | 350.29 (1504558.67) | 7.43 (7.38) | 175.64 (167.32) |
| Skin and subcutaneous tissue disorders | Photosensitivity reaction | 19 | 1501.15 (1408.67-1599.71) | 703.02 (1046449.53) | 8.3 (8.22) | 352.01 (330.32) |
|  | Hyperhidrosis | 15 | 1486.31 (1423.59-1551.79) | 177.54 (1725771.18) | 6.47 (6.44) | 89.27 (85.5) |
| Vascular disorders | Hypertension | 82 | 1513.38 (1451.83-1577.54) | 105.02 (1816765.29) | 5.73 (5.7) | 53.01 (50.85) |
|  | Hot flush | 23 | 1499.08 (1428.12-1573.57) | 347.59 (1509626.59) | 7.42 (7.37) | 174.29 (166.04) |
|  | Thrombosis | 11 | 1489.28 (1423.01-1558.63) | 258.16 (1623012.97) | 7 (6.96) | 129.58 (123.81) |
|  | Blood pressure fluctuation | 8 | 1492.25 (1417.33-1571.13) | 433.86 (1394714.53) | 7.72 (7.66) | 217.43 (206.51) |

**Supplementary Table S1.** Signal strength of reports of niraparib (excluding consumer reports) at the Preferred Term (PT) level in FAERS database.

^*^Emerging findings of niraparib associated AEs from FAERS database. ROR, reporting odds ratio; CI, confidence interval; PRR, proportional reporting ratio; χ, chi-squared; IC, information component; IC025, the lower limit of 95% CI of the IC; EBGM, empirical Bayesian geometric mean; EBGM05, the lower limit of 95% CI of EBGM.

| Algorithms | Equation | Criteria |
| --- | --- | --- |
| ROR | ROR=ad/b/c | lower limit of 95% CI>1, N≥3 |
|  | 95%CI=e^ln(ROR)±1.96(1/a+1/b+1/c+1/d)^0.5^ |  |
| PRR | PRR=a(c+d)/c/(a+b) | PRR≥2, χ^2^≥4, N≥3 |
|  | χ^2^=[(ad-bc)^2](a+b+c+d)/[(a+b)(c+d)(a+c)(b+d)] |  |
| BCPNN | IC=log_2_a(a+b+c+d)(a+c)(a+b) | IC025>0 |
|  | 95%CI= E(IC) ± 2V(IC)^0.5 |  |
| MGPS | EBGM=a(a+b+c+d)/(a+c)/(a+b) | EBGM05>2 |
|  | 95%CI=e^ln(EBGM)±1.96(1/a+1/b+1/c+1/d)^0.5^ |  |

**Supplementary Table S2. Four statistical procedures used for signal detection.**

Equation: a, number of reports containing both the target drug and target adverse drug reaction; b, number of reports containing other adverse drug reaction of the target drug; c, number of reports containing the target adverse drug reaction of other drugs; d, number of reports containing other drugs and other adverse drug reactions. 95%CI, 95% confidence interval; N, the number of reports; χ2, chi-squared; IC, information component; IC025, the lower limit of 95% CI of the IC; E(IC), the IC expectations; V(IC), the variance of IC; EBGM, empirical Bayesian geometric mean; EBGM05, the lower limit of 95% CI of EBGM.
